# Urotensin-II Immunoreactivity in Normolipidemic and Hyperlipidemic New Zealand White Rabbits Following Balloon Angioplasty and Stenting

**Published:** 2007-03

**Authors:** Nicolas Bousette, Fazila Chouiali, Eliot H. Ohlstein, Stephen A. Douglas, Adel Giaid

**Affiliations:** 1 *Division of Cardiology, Montreal General Hospital, McGill University Health Center, Montreal, Quebec, Canada;*; 2 *The Cardiovascular and Urogenital-CEDD, GlaxoSmithKline, King of Prussia, PA, USA*

**Keywords:** endothelium, immunohistochemistry, vascular injury, peptide

## Abstract

Treatment for symptomatic atherosclerosis is being carried out by balloon mediated angioplasty, with or without stent implantation, more and more frequently. Although advances with the development of drug eluting stents have improved prognosis, restenosis is still the most limiting factor for this treatment modality. Urotensin-II (UII), a small pleiotropic vasoactive peptide is increasingly being recognized as a contributory factor in cardiovascular diseases. We qualitatively evaluated UII immunoreactivity (IR) in three models of balloon angioplasty mediated restenosis. Specifically, we performed balloon angioplasty in the ilio-femoral arteries of New Zealand White Rabbits (NZWR) fed either a normal chow or high fat diet. In addition, UIIIR was also assessed in stent implanted abdominal aortae of NZWR fed a high fat diet. UII was constitutively expressed in the endothelium of all arterial segments evaluated. Abundant expression of UII was associated with lesion progression, particularly in myointimal cells, and less so in medial smooth muscle cells (SMC). The strongest UII-IR was observed in foam cells of animals fed a high fat diet. We demonstrate abundant expression of UII in regenerating endothelial cells and myointimal cells in vascular lesions following balloon mediated angioplasty and stent implantation in both animals fed a normal chow and high fat diet.

## INTRODUCTION

Percutaneous coronary angioplasty, a modality for the treatment of atherosclerosis, is being employed more frequently and in more difficult cases every day. Although this procedure has been dramatically improved with the use of stents, especially drug eluting stents, restenosis is still the most limiting factor to its ultimate success. Indeed, although stent implantation reduces the risk of restenosis by 10 folds compared with balloon angioplasty alone (1), more than 90% of the late lumen loss after stent implantation is caused by neointimal formation (2). Restenosis is considered an arterial healing response that is initiated by imigration and proliferation of SMCs in the intimal layer with subsequent elaboration of the extracellular matrix (3-4).

Human UII, a potent vasoactive cyclic undecapeptide, has recently been the focus of a number of cardiovascular studies. UII is a vasoactive factor inducing vasoconstriction of some arteries such as the rat aorta, while inducing endothelial mediated vasodilation of other vessels (5-6). UII induced proliferation of endothelial cells, smooth muscle cells, cardiac fibroblasts as well as hypertrophy of cardiac myocytes (7-10). UII expression is increased in failing human hearts as well as in plasma of patients with heart failure (11-12). Similarly, UII is increased in atherosclerotic coronary arteries as well as in plasma of patients with documented coronary atherosclerosis (13).

Recently, we have demonstrated that UII is elevated in human aortic and coronary atherosclerosis (14-15). These findings are supported by Maguire *et al*. (2004) whom also demonstrated UII immunoreactivity in coronary atherosclerosis an in failed saphenous veins (16). We also recently demonstrated that UII has a role in a model of angioplasty mediated carotid artery restenosis in the rat (17). In the latter study, UII expression was significantly increased in the neointima of a rat carotid artery following balloon mediated angioplasty, and blockade of UII signaling, using a selective UT receptor antagonist (SB-611812), significantly attenuated intimal thickening in the same model. Thus, indicating that UII may have a role in SMC proliferation in the neointima of injured arteries. In support of this, UII was shown to have potent mitogenic activity on cultured SMCs from both rat (8) and rabbit (18). Therefore, the aim of the present study was to assess the arterial expression of U-II in normolipidemic and hyperlipidemic New Zealand White Rabbits following balloon angioplasty and stenting.

## MATERIALS AND METHODS

Male New Zealand White rabbits (NZWR) (age 3 months) weighing 2-4 kg were studied (n=12-16 per group, n=2-4 per time point). Animals were divided into the following groups: normal chow diet fed rabbits following balloon angioplasty of iliofemoral arteries, high fat diet fed rabbits following balloon angioplasty of iliofemoral arteries, and rabbits on high fat diet with stented abdominal aorta.

All animal work was performed in accordance with institutional guidelines, and in compliance with the guide for the care and use of laboratory animals, published by the national institutes of Health (NIH publication 85-23 revised 85). Male NZWR were housed individually and fed a normal chow or high fat /cholesterol diet containing 2.5% peanut oil and 0.5% cholesterol (TD 98263, Harlan Teklad) ~125 g/day. The animals were adjusted to the high fat diet over 7 days. After 2 weeks on high fat diet, balloon angioplasty (inflation and drag 3X) was performed in the iliac portion of the left femoral artery (Day T0).

### Ilio-Femoral angioplasty and abdominal aortic stenting

Following induction of general anesthesia, the animal was placed in dorsal recumbency and its abdominal and ventral femoral areas were shaved. The animal was scrubbed and draped for aseptic surgery, and the drape was cut to expose a muscular branch of the left femoral artery. A 2 cm incision was created next to the muscular branch, and the muscular artery was isolated by blunt dissection. The distal end of the vessel was ligated using 6-0 prolene and a loosely knotted 6-0-prolene suture was placed proximal to the introduction site of the catheter.

For balloon angioplasty of the ilio-femoral arteries, a moistened 3.0 french Fogarty balloon catheter (Baxter) was evacuated of air and filled with 0.2 ml of saline. The balloon was checked for leaks and then deflated. Using a sufficient amount of saline to keep the catheter and introduction site moist, the vessel was cut and the catheter introduced into the artery. The balloon catheter was inserted 8-9 cm and pulled back, with a twisting motion, approximately 7-8 cm. To create adequate tension within the vessel, the balloon was inflated 0.09-0.11 ml while being pulled through the artery. This denuding procedure was repeated three times. The muscular artery was ligated as the catheter was withdrawn. The incision site was rinsed with saline and the sub-dermal layer closed using a continuous pattern with 6-0 prolene. Interrupted sutures of 4-0 prolene were used for dermal closure. The contralateral artery from each animal was used as naïve arteries.

For abdominal aortic, stenting a sterile stent (Nirol, Vacco Industries, Part # M1110) was inserted into the end of a section of PE50 tubing. A metal stylet was inserted into the opposite end of the tubing and advanced to the stent. The stent and catheter were moistened with saline and the upper end of the catheter and stylet clamped with mosquito forceps. The femoral artery was cut and the stent-catheter introduced 10 cm into the abdominal aorta. The proximal tie was tightened, the mosquito forceps were released, and the stent was deployed by gently pushing the stent out of the catheter with the stylet. Once the stent is deployed, the catheter was removed and the femoral artery branch was ligated. The incision site was rinsed with saline and the sub-dermal layer closed using a continuous pattern with 6-0 prolene. Interrupted sutures of 4-0 prolene were used for dermal closure. Vet-bond was applied to the closed incision if necessary immediately following surgery; ultrasound was done on each rabbit to confirm proper deployment of the stent in the distal abdominal aorta.

### Immunohistochemistry

Immunohistochemical staining for UII and UT was performed using the avidin–biotin peroxidase method as previously described (19). Monoclonal antisera to human U-II were generated (by T. Wattam, Department of Immunology, GlaxoSmithKline, Harlow, UK) as previously described (11). The types of cells were verified in a limited number of adjacent sections using appropriate markers as shown previously (14-15).

Paraffin sections (5μm) were immunostained with a monoclonal antiserum to human U-II. Sections were counterstained with hematoxylin. Negative control sections included immunoabsorption of the antisera with their own antigens, and the use of non-immune serum in place of the primary antibody.

## RESULTS

### Expression of U-II in NZWR on Normal Chow Diet after iliofemoral balloon angioplasty

**Figure 1:**
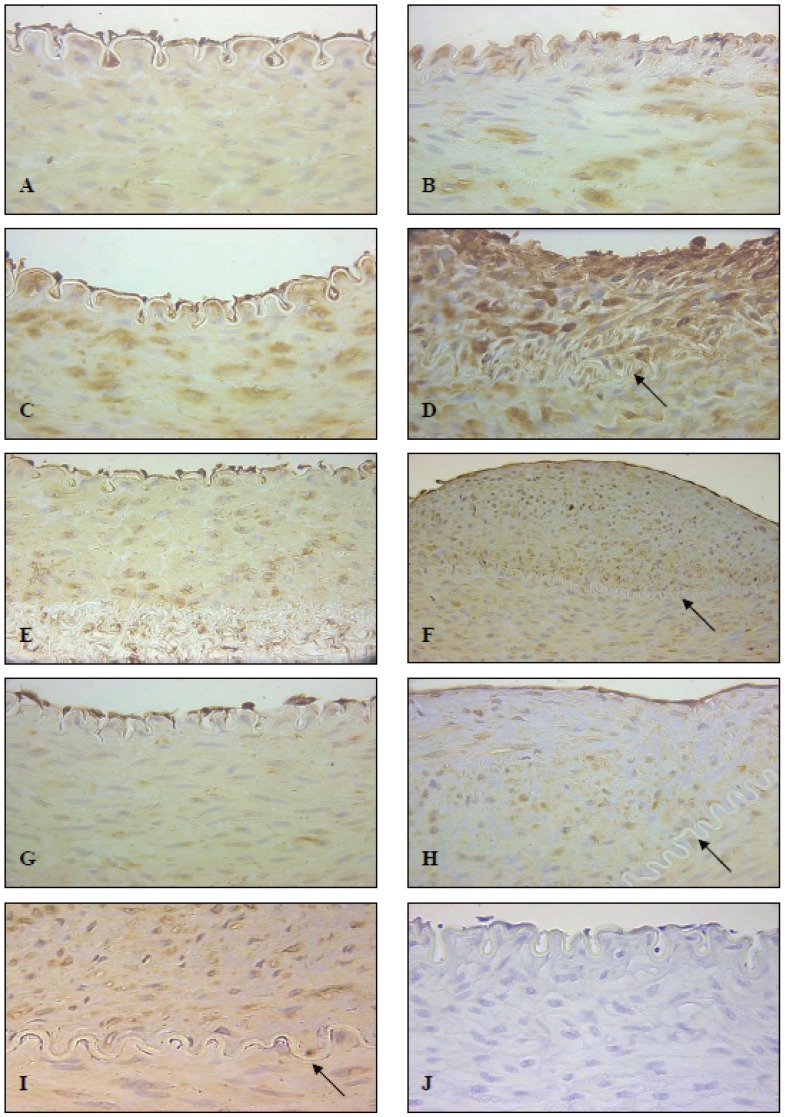
Expression of U-II in NZWR on Normal Chow Diet after iliofemoral balloon angioplasty. Panel A demonstrates a naïve artery one day postangioplasty (400× magnification). Panel B demonstrates the injured artery 1 day post-angioplasty (400× magnification). Panel C demonstrates a naïve artery 7 days post-angioplasty (400× magnification). Panel D demonstrates the injured artery 7 days post angioplasty (arrow indicates internal elastic lamina; 400× magnification). Panel E demonstrates a naïve artery 14 days post-angioplasty (400× magnification). Panel F demonstrates the injured artery 14 days post-angioplasty (arrow indicates internal elastic lamina; 200× magnification). Panel G demonstrates a naïve artery 28 days post angioplasty (400× magnification). Panel H demonstrates an injured artery 28 days post-angioplasty (arrow indicates internal elastic lamina; 400× magnification). Panel I demonstrates increased UII-IR in the myointimal cells of the neointima relative to that of the medial SMCs in an injured artery 28 days post-angioplasty (400× magnification). Panel J demonstrates a negative control section showing no non-specific UII-IR (400× magnification).

The naïve/control arteries showed moderate expression of UII in the vascular endothelial cells, while expression in the media was weak to moderate (Fig 1A). One day post-angioplasty the contralateral injured iliofemoral arteries of NZWR on normal diet expectedly lacked an endothelium. Interestingly, most of these arterial segments displayed strong IR directly beneath the internal elastic lamina, while medial SMC IR was moderate to strong (Fig 1B). Three days post-angioplasty, the uninjured naïve artery showed no change in the expression of U-II with endothelial cells exhibiting relatively moderate IR while medial SMCs exhibited weak UII IR. The injured contralateral arteries continued to lack an endothelium, while the sub-laminal IR had dissipated three days post-angioplasty (data not shown). Furthermore, the medial SMC IR in these arterial segments was only weak to moderate.

In naïve arteries of animals 7 days post-angioplasty, medial SMCs showed strong UII IR in addition to the prominent EC staining (Fig 1C). At 7 days post-angioplasty, injured arteries displayed regions of intimal thickening, with re-endothelialization. The endothelial cells demonstrated strong IR, as did inflammatory and myointimal cells of the neointima (Fig 1D). Medial SMC IR of injured arteries was also moderate to strong 7 days post-angioplasty.

At 14 days post-angioplasty, naïve arteries demonstrated relatively strong endothelial cell IR while medial SMC staining was moderate (Fig 1E). Injured arteries showed strong endothelial cell staining in addition to a much more prominent neointima, which exhibited moderate to strong UII IR (Fig 1F). At 28 days post-angioplasty naïve arteries displayed very strong EC IR and moderate medial IR (Fig 1G). The contralateral injured arteries displayed substantial intimal thickening with the presence of very strong UII-IR in both the endothelial and myointimal cells within the neointima (Fig 1H). Medial IR was moderate to strong in these segments. UII-IR was consistently stronger in the myointimal cells of lesions than in native medial SMCs (Fig 1I). UII immunoreactivity was absent in negative control sections (Fig 1J).

### Expression of U-II in NZWR on High Fat Diet after iliofemoral balloon angioplasty

**Figure 2:**
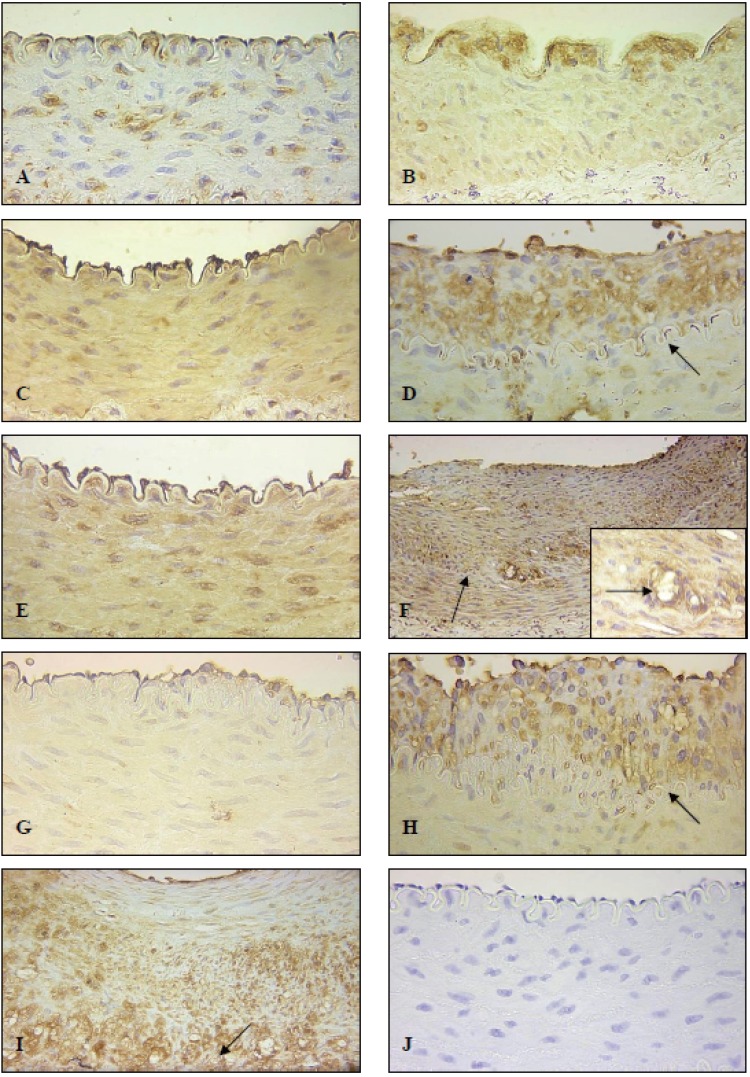
Expression of U-II in NZWR on high fat diet after iliofemoral balloon angioplasty. Panel A demonstrates a naïve artery one day post-angioplasty (400× magnification). Panel B demonstrates the injured artery 1 day post-angioplasty (400× magnification). Panel C demonstrates a naïve artery 7 days post-angioplasty (400× magnification). Panel D demonstrates the injured artery 7 days post angioplasty (arrow indicates internal elastic lamina; 400× magnification). Panel E demonstrates a naïve artery 14 days post-angioplasty (400× magnification). Panel F demonstrates the injured artery 14 days post-angioplasty (arrow indicates internal elastic lamina; 200× magnification). Panel F insert is a higher magnification of panel F, showing strong UII-IR associated with foam cells. Panel G demonstrates a naïve artery 28 days post angioplasty (400× magnification) with a very thin neointimal layer. Panel H demonstrates neointima of naïve artery 28 days post-angioplasty (arrow indicates internal elastic lamina; 400× magnification). Panel I demonstrates an injured artery 28 days post-angioplasty (arrow indicates internal elastic lamina; 200× magnification). Panel J demonstrates a negative control section showing no non-specific UII-IR (400× magnification).

One day post-angioplasty the naïve artery demonstrated moderate UII-IR in both the endothelial and medial SMC cells (Fig 2A). UII-IR in the injured artery one day post-angioplasty was localized predominantly to the subintima (Fig 2B), which was similarly noted in animals on normal chow diet one day post-angioplasty. Expectedly, the endothelium was absent in these arteries.

In concordance with observations in arterial segments of animals on normal chow diet, animals on high fat diet exhibited elevated UII expression in both naïve and injured arteries seven days post-angioplasty. Naïve arteries demonstrated moderate to strong UII-IR in the tunica media, while the endothelium demonstrated relatively strong UII-IR (Fig 2C). The injured arteries on the other hand exhibited strongest UII-IR in the neointima, with lesser IR in the media (Fig 2D). These latter arteries also demonstrated re-endothelialization with strong IR.

At 14 days post angioplasty, naïve arteries exhibited moderate to strong UII-IR in both the endothelium and in the media (Fig 2E). Contralateral injured arteries exhibited substantial intimal thickening with abundant UII-IR in foam cells in the abluminal side of the neointima (Fig 2F). The tunica media also exhibited relatively strong UII-IR in these arteries.

At 28 days post-angioplasty, Naïve arteries exhibited moderate endothelial and weak medial IR (Fig 2G). A relatively small neointima was observed in this artery demonstrating the atherogenic potential of the high fat diet in these animals. Similarly to injured vessels, the neointima of this naïve artery demonstrated strong UII-IR (Fig 2H). The contralateral injured arteries exhibited very large intimal thickenings with very strong IR in the endothelial, neointimal and medial layers (Fig 2I). Consistently, strongest UII-IR was noted in the outer neointima in association with foam cells.

### Expression of U-II in NZWR on High Fat Diet after Aortic stenting

**Figure 3:**
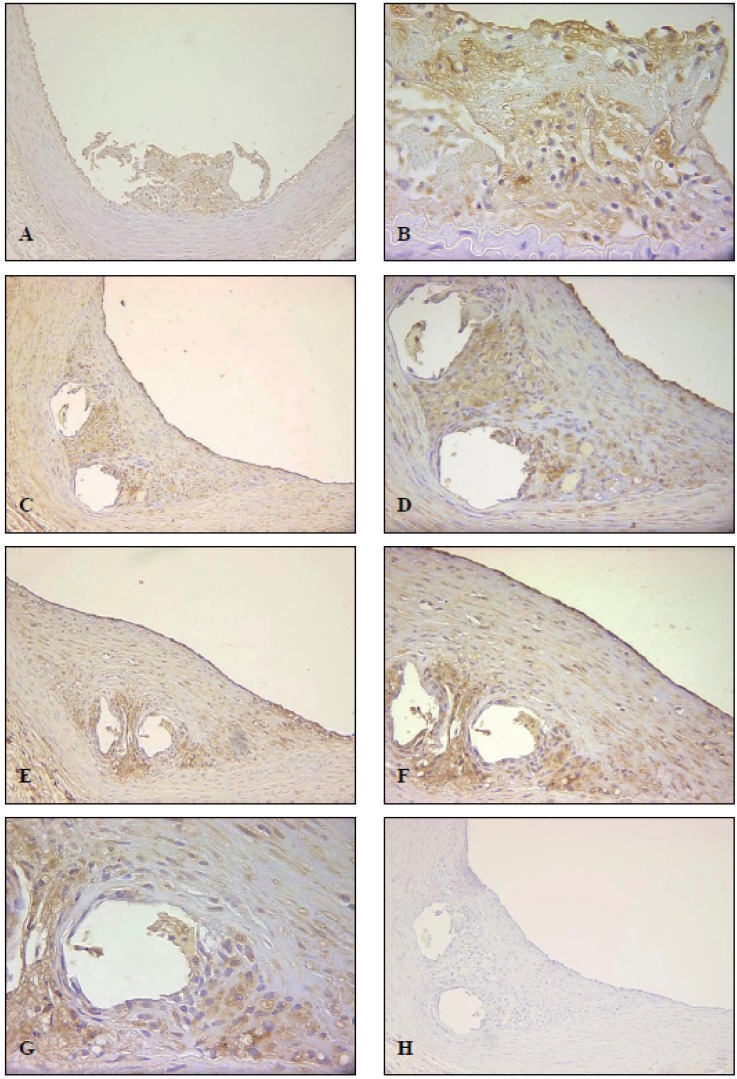
Expression of U-II in NZWR on high fat diet after aortic stenting. Panel A shows a section of the stented abdominal aorta 7 days post-procedure (100× magnification). Panel B is higher magnification of panel A demonstrating diffuse UIIIR in a disorganized thrombus/lesion (400× magnification). Panel C shows a lesion in a stented aorta 14 days postprocedure (100× magnification). Panel D is higher magnification of panel C (200× magnification). Panel E shows a large lesion in a stented aorta 28 days post-procedure (100× magnification). Panel F is higher magnification of panel E (200× magnification). Panel G demonstrates large lesion with strong UII-IR in both foam cells and lesion myoinitimal cells of a stented aorta 28 days post-procedure (400× magnification). Panel H demonstrates a negative control section showing no non-specific UII-IR (100× magnification).

Sections of NZWR aorta on high-fat diet one week following stenting exhibited moderate expression of U-II in endothelial cells while moderate and diffuse UII expression was observed in the thrombus/lesion surrounding the stent (Fig 3A). These early lesions which presented as disorganized thrombi (Fig 3B), projected into the lumen of the aorta surrounding the stent, while sub-lesion media was unaltered indicating an absence of vascular remodeling at this early time point. Medial IR was weak at this time point.

At two weeks, U-II expression remained strong in endothelial cells while it increased within lesions. In fact, both myointimal and foam cells showed strong UII-IR in these animals (Fig 3C & D). Interestingly, vascular remodeling was evident at this time point as lesions no longer projected into lumen and sub-lesion media showed substantial atrophy (Fig 3C). Medial IR was slightly elevated in these animals (Fig 3C). Interestingly, UII-IR was consistently stronger in the outer vs. inner neointimal layer, in association with foam cells (Fig 3C-G).

At 4 weeks post-stenting there was an evident increase in lesion size/number (Fig 3E). The endothelial IR remained strong at this time point. The large lesions demonstrated strong UII-IR in both foam cells and lesion myoinitimal cells (Fig 3F-G). The medial IR was moderate to strong in these animals. Negative control sections from all experimental groups showed no expression of U-II (Fig 3H).

## DISCUSSION

In the present study, we qualitatively characterized UII protein immunoreactivity in NZWR on normal chow and high fat diets following angioplasty in the ilio-femoral artery. Furthermore, we also qualitatively assessed UII immunoreactivity in NZWR on a high fat diet following abdominal aortic stenting. Therefore, the present study aimed at evaluating UII expression in a qualitative fashion, across a broad spectrum of vasculopathies, at both early and late time points after percutaneous interventions. Although the study included a large number of animals, the numerous study groups allowed for only an n=4 animals per group which was not appropriate for accurate quantifications. The results showed that there was nominal U-II expression in the endothelium of naïve arteries, while the expression of the peptide in the vascular endothelium and underlying cells of the thickened intima intensified with the progression of the lesion. In addition, UII expression was more prominent in myointimal cells of vascular lesions than in medial SMCs, but was strongest in foam cells of animals fed a high fat diet. Indeed, UII immunoreactivity was more pronounced in arteries from animals fed a high fat diet in large part due to the strong foam cell staining which was absent in animals fed a normal chow diet.

There are several key points that can be appreciated from the qualitative demonstration of UII-IR following angioplasty. Firstly, UII was consistently expressed in the endothelium of both ilio-femoral arteries and abdominal aorta of animals on either normal chow or high fat diet, indicating that the endothelium constitutively expresses UII. This is in agreement with our previous report which showed UII immunoreactivity in normal and diseased human aorta (14).

In the acute setting, that is 1 day following angioplasty, UII immunoreactivity was strong just below the internal elastic lamina in angioplasty injured arteries (in animals on either normal chow or high fat diet), suggesting a direct effect of the injury response on UII expression. Interestingly, this sub-laminal UII expression had dissipated after three days. However, at 7 days post angioplasty, UII-IR was very strong in injured arteries demonstrating that the stimulus for UII expression is biphasic with an acute up-regulation (noted on day 1) and a chronic phase (noted from day 7-28). In addition, it was interesting to note that that UII-IR was also elevated in naïve arteries (7-days post-angioplasty), which suggests that a circulating humoral factor, induced by arterial injury, has a systemic effect on UII expression in the vasculature.

Whether the SMCs involved in lesion formation are dedifferentiated vascular SMCs or are derived from phenotypically abberant SMCs is still a matter of serious debate. However, there is strong evidence that there is in fact a heterogeneous pool of SMCs resident in the media (20). This heterogeneous pool is believed to consist of at least two phenotypically different vascular SMC. These include the differentiated long fusiform or spindle shaped cells which are positive for smooth muscle myosin heavy chain and SMC-actin, and the epitheloid spherical cells which are negative for smooth muscle myosin heavy chain but positive for SMC-actin. The former are serum insensitive and have poor hyperplasic activity while the latter have demonstrated serum responsive proliferation in culture (21). Indeed, there is evidence that these epitheloid-type SMCs exhibiting a proliferative/synthetic phenotype are predominant in restenotic lesions, whereas SMCs of the media are predominantly differentiated spindle shaped and exhibit a contractile phenotype (21). Interestingly, UII immunoreactivity was consistently strong in all vascular lesions (intimal thickenings and atherosclerotic plaques). This was apparent from early stage lesions, to later time points in which there was a well developed neointima or large fibrofatty atherosclerotic plaque. Moreover, UII expression was consistently stronger in the myointimal cells of lesions than in medial SMCs. Therefore, this may suggest that UII expression is favored in epitheloid SMCs with a proliferative/synthetic phenotype compared to spindle shaped contractile SMCs of the media. In this regard, it is interesting to note that UII has demonstrated pro-fibrotic effects including induction of collagen synthesis in both cardiac fibroblasts and endothelial cells (7, 10). Whether this pro-fibrotic activity extends to SMCs, especially those with a synthetic phenotype, and whether UII has a role in extracellular matrix deposition in the neointima needs to be determined.

The prominent UII immunoreactivity associated with myointimal cells and medial SMCs is interesting in light of several studies demonstrating UII as a SMC mitogen (8, 18). Therefore, based on its prominent expression in lesions and its potent mitogenic actions, UII is likely an active contributor in the pathological sequelae following balloon mediated angioplasty. This is supported by our recent study which demonstrated that UII blockade using a selective UII receptor antagonist, SB-611812, significantly attenuated intimal thickening in a rat model of balloon angioplasty mediated restenosis.

In animals fed a high fat diet it was apparent that UII expression was strongest in foam cells. This was evident in both ilio-femoral arteries and abdominal aorta of animals on the high fat diet but was expectedly absent in those animals on a normal chow diet. This is especially interesting in light of a recent study by Watanabe *et al*. which demonstrated that UII induced foam cell formation by increasing ACAT activity and expression (22). Furthermore, they showed that UII increased cholesterol-ester loading induced by acetylated-LDL. Therefore, we provide further supportive evidence of a role for UII in foam cell formation.

In summary, we show that UII expression is associated with endothelial, myointimal, and foam cells of restenotic lesions in both normo- and hyperlipidemic rabbits following either balloon angioplasty or stent-mediated restenosis. Future studies using a selective UT receptor antagonist like the one we have previously used, or deletion and/or overexpression the UII and UT genes would determine the exact role the urotensin system plays in these disease modalities.
